# Oral Exposure to Nylon-11 and Polystyrene Nanoplastics During Early-Life in Rats

**DOI:** 10.3390/nano15060465

**Published:** 2025-03-19

**Authors:** Ninell P. Mortensen, Maria Moreno Caffaro, Archana Krovi, Jean Kim, Scott L. Watson, Rodney W. Snyder, Purvi R. Patel, Timothy R. Fennell, Leah M. Johnson

**Affiliations:** RTI International, 3040 E. Cornwallis Road, Research Triangle Park, Durham, NC 27709, USAmmoreno@rti.org (M.M.C.); akrovi@rti.org (A.K.); jeankim@rti.org (J.K.); slwatson@rti.org (S.L.W.); rsnyder@rti.org (R.W.S.); fennell@rti.org (T.R.F.)

**Keywords:** nanoplastics, nylon, polyamide, polystyrene, early-life exposure, cardiac assessment, metabolomic analysis, oral exposure

## Abstract

A critical knowledge gap currently exists regarding the potential risks of exposure to nanoplastics (NPs), particularly early in life during key stages of growth and development. Globally abundant plastics, polyamide (nylon) and polystyrene (PS), exist in various products and have been detected in food and beverages as small-scale plastics. In this study, we evaluated how early-life exposure to NPs affects key biological metrics in rat pups. Male and female animals received an oral dose (20 mg/kg/day) of nylon-11 NPs (114 ± 2 nm) or PS NPs (85 ± 1 nm) between postnatal day (PND) 7 and 10. The results showed slight differences in the ratio of liver weight to body weight for male rat pups exposed to PS NPs. Cardiac performance and levels of neurotransmitters and related metabolites in brain tissue showed no differences between animals exposed to NPs and controls. The endogenous metabolite profile in plasma was altered by oral administration of NPs, suggesting perturbation of metabolic pathways involved in amino acid and lipid metabolism. This study explored the biological impacts of oral NP exposure early in life, supporting the need for continued investigations into the potential health effects from exposure to NPs.

## 1. Introduction

The global ubiquity of plastics has resulted in the presence of microplastics (MPs) and nanoplastics (NPs) in the environment [1,2], foodstuffs [3,4,5], and drinking water [6,7,8]. Reports of plastics in human blood [9], thrombi [10], lung tissue [11], placenta [12,13], breast milk [14], reproductive tissues [15,16], and sputum [17] raise critical questions about the effects of small-scale plastics on human health. Moreover, the identification of MPs in human urine [18] and stool [19,20], along with links between MPs and irritable bowel syndrome (IBS) in humans [21], further illustrate the potential impacts of oral exposure to these materials. The possible influence of nanoscale plastics on human health is particularly significant, owing to the ability of nanomaterials to readily enter cells through mechanisms such as endocytosis [22,23]. The physicochemical properties of nanomaterials can result in either innocuous or toxicological outcomes in biological systems [24]. For example, many biocompatible nanoformulations are safe and beneficial for biomedical applications including drug delivery, bioimaging, and tissue engineering [25,26,27]. Conversely, other nanomaterials, including some metallic and carbon-based engineered nanomaterials (ENMs), show toxicological effects in mammalian systems [28,29,30,31]. NPs are categorized as a unique class of particles separate from ENMs [32] and the possible biological effects from exposure to NPs are largely unknown.

The abounding questions surrounding NPs and human health are particularly critical for populations with a heightened vulnerability to environmental contaminants. For example, early-life exposures to environmental toxins can affect critical stages of development and growth and potentially increase the risks of diseases later in life [33,34,35,36]. Early-life exposures can occur during the prenatal period, but also postnatally when certain organ systems are still developing [37,38,39]. To date, studies involving exposure to sub-micron plastics have predominantly used well-characterized polystyrene (PS) particles [40,41,42,43,44,45,46,47,48,49,50,51,52]. For example, pulmonary exposure of pregnant Sprague Dawley rats to PS NPs (rhodamine-labeled, 20 nm) resulted in the translocation of particles to fetal and placental tissues [46]. In another study, the oral administration of PS NPs to pregnant mice resulted in the progeny exhibiting neurophysiological abnormalities and cognitive impairment in a gender-specific manner [49]. The exposure of adult mice to PS NPs (80 nm) during pregnancy showed sex-specific intestinal toxicity in the offspring [51]. An increased risk of metabolic disorders occurred in the offspring of mice orally exposed to polystyrene beads (0.5 µm or 5 µm) during gestation [52].

Studies with PS NPs are valuable for understanding the effects of small-scale plastics; however, the compositions of global commodity plastics are substantially more diverse, with some examples including polypropylene, polyethylene, and polyamide. Moreover, the heterogeneous origins of MPs and NPs, combined with diverse environmental exposures, can ultimately result in a plethora of physicochemical properties with different shapes, sizes, and compositions. The origins of MPs and NPs are typically categorized as primary or secondary sources. Primary sources include intentionally fabricated particles, with applications such as personal care products [53,54], or starting materials for parts manufacturing [55]. Secondary sources result from the reduction in larger-sized plastic after exposure to extraneous perturbations, such as mechanical stress, ultraviolet (UV) irradiation, or solvent exposure [56]. Attaining a broad understanding of how small-scale plastics may affect biological systems and ultimately human health will require the evaluation of particles with various polymeric compositions and physicochemical properties.

With an estimated global production of 6.7 million metric tons in 2023 [57], nylon is a commodity plastic with widespread use in film packaging, textiles, molded parts, and biomedical applications [58]. The term nylon encompasses a class of polyamide thermoplastics that differ based on the chemical arrangements within the backbone of the polymer. As synthetic materials, nylons have existed for nearly a century with an early example including nylon-6,6, which was developed by Wallace Hume Carothers in 1935 as an entirely synthetic fabric [59]. Another prominent example is nylon-11, which shows low moisture absorption and is used for many marine, automotive, and agricultural applications [60]. Despite the ubiquity of nylons in modern society, along with the detection of small-scale nylon particulates in food items and beverages [61,62,63,64], the effects from oral exposure to nylon nanoparticles on biological systems is largely unknown. A few examples exist describing the preparation of nanoparticles of nylon-11 with accompanying evaluations in mammalian cells [65,66]. Additionally, nylon-based nanofibers have also been used as cellular scaffolds in applications such as guiding neurite growth [67] and supporting the growth of bone tissue [68].

Currently, the effects of oral exposure to MPs and NPs during the early stages of life are largely unknown, and critical questions exist regarding potential toxicological responses and effects on developmental processes. This study explores the biological impacts that result from oral exposure to nylon NPs in vivo. With an emphasis on early-life exposure, this study tests the effects of oral exposure to nylon-11 NPs and PS NPs on Sprague Dawley rats, aged 7–10 postnatal days (PNDs). The findings support continued advancements in understanding the effects of NPs on biological systems and ultimately human health.

## 2. Materials and Methods

### 2.1. Chemicals and NPs

PS NPs (0.05–0.1 µm diameter) were purchased from Spherotech, Inc. (catalog number PP-008-10, Lake Forest, IL, USA). The PS NPs were supplied as an aqueous suspension in 0.02 w/w% sodium azide and were not washed prior to use in this study. For the fabrication of nylon-11 NPs, hexafluoroisopropanol (HFIP; Sigma-Aldrich Catalog Number 105228) and nylon-11 (Sigma-Aldrich Catalog Number 181153) were purchased from Sigma-Aldrich (St. Louis, MO, USA). The components for the neurotransmitter analysis were purchased from Sigma-Aldrich: citric acid, the internal standard 3,4-dihydroxybenzylamine (DHBA), sodium phosphate (monobasic) NaH_2_PO_4_, EDTA, octanesulfonic acid, dopamine (DA), dihydroxyphenylacetic acid (DOPAC), homovanillic acid (HVA), 5-hydroxyindole-3-acetic acid (5-HIAA), norepinephrine (NE), and serotonin (5-HT).

### 2.2. Fabrication of Nylon-11 NPs

The fabrication of nylon-11 NPs was based on a previously published procedure [65]. Briefly, a solution was prepared by combining 0.35 g of nylon-11 with 20 mL of HFIP in a 40 mL scintillation vial. The nylon-11 solution was added dropwise (10 mL, 1 mL/min) to ultrapure deionized water (75 mL, 18.2 MΩ·cm resistivity) via a syringe pump (Model # NE-300, New Era Pump Systems, Inc., Farmingdale, NY, USA). Residual HFIP was removed from the solution of nylon-11 NPs using rotary evaporation under vacuum at 60 °C. When the volume reduced to ~30 mL, additional ultrapure deionized water (~75 mL) was added to the flask and the rotary evaporation continued. This process was repeated for a total of 5 times.

### 2.3. Characterization of NPs

#### 2.3.1. Dynamic Light Scattering (DLS) and Zeta Potential

The NPs were characterized with the Zetasizer Nano ZS (Malvern Instruments, Malvern, UK) equipped with a He-Ne laser (633 nm). A non-invasive backscatter method was used with a scattering angle of 173°. The hydrodynamic diameter (D_H_), polydispersity index (PDI), and zeta potential of plastic nanoparticles were calculated with the instrument software (Zetasizer DTS, version 7.13).

#### 2.3.2. ^19^F Nuclear Magnetic Resonance Spectroscopy (^19^F-NMR)

^19^F-NMR was used to evaluate residual HFIP in the solution of nylon-11 NPs. The samples were mixed with D_2_O at 10% (*v*/*v*) and characterized using a Varian Unity Inova 500 mHz NMR (Palo Alto, CA, USA) with a Nalorac Cryogenics Corporation dedicated H-F observed probe (Martinez, CA, USA). The total recycling time was 8 s. To calibrate and quantify the remaining HFIP, an external reference standard was used along with the Agilent VnmrJ ver. 4.2 software (Santa Clara, CA, USA) with a limit of detection of 0.02 mM. 

#### 2.3.3. Scanning Electron Microscopy (SEM)

Samples of nanoparticles were deposited on an aluminum stub covered with double-sided copper tape. The nanoparticles were sputter-coated with ~13 nm of Au/Pd using a Cressington 108 Auto sputter coater (Cressington Scientific Instruments Ltd., Watford, UK). SEM images were obtained with a Hitachi S-4700 cold cathode field emission SEM (Hitachi High-Tech, Schaumburg, IL, USA).

#### 2.3.4. Evaluation of Endotoxin

To detect and quantify endotoxins, a pyrochrome test kit (Associates of Cape Cod Inc., East Falmouth, MA, USA) was utilized [69]. PS NPs and nylon-11 NPs were diluted to a concentration of 1.0 mg mL^−1^ with endotoxin-free limulus amebocyte lysate (LAL) reagent water (Associates of Cape Cod Inc., East Falmouth, MA, USA). The LAL reagent water was also used for testing the supernatant obtained from the PS NPs and nylon-11 NPs. To confirm that the NPs did not interfere with the assay, positive product controls (PPCs) that contained a final concentration of 0.5 endotoxin units (EUs) per mL were evaluated in parallel at the same concentration. For the tested concentrations, no interference between the NPs and the assay was detected.

#### 2.3.5. Concentration of NPs

A 1 mL aliquot of NPs was transferred to a tared 2 mL Eppendorf tube and placed in a SpeedVac concentrator (Labconco SpeedVac SC 100, Kansas City, MO, USA) at 40 °C under a vacuum for 6 h. The dry weight of the NPs was determined by weighing the tube and the concentration of NPs in the original solution, calculated as weight/volume.

### 2.4. In Vivo Rat Study

#### 2.4.1. Housing and Dose Administration

Sprague Dawley rat dams and their standardized litters of five male and five female pups aged 1 PND were sourced from Charles River Laboratories (Raleigh, NC, USA). The handling and care of the animals complied with the Guide for the Care and Use of Laboratory Animals [70] and was approved by the Institutional Animal Care and Use Committee (IACUC) of Mispro Biotech, Research Triangle Park, NC, USA. Rat dams and their litters were housed in individual polycarbonate cages and fed LabDiet 5058 Breeder Diet (LabDiet, Durham, NC, USA) and Durham City (NC) water, provided ad libitum. Each dosing group comprised three litters, totaling 15 female and 15 male pups (Figure S1). On PND 4, litters were normalized to contain 5 male and 5 female pups. Of note, one male from the nylon-11 NP dosing group was removed from the study on PND 10 due to low body weight and insufficient growth. The reason for the low body weight of this one animal is unknown. The room that housed the animals was maintained at approximately 72 ± 3 °F, 30–70% relative humidity and light/darkness cycled at 12-h intervals. Male and female pups received four daily consecutive doses between PND 7–10 of 20 mg/kg/day of nylon-11 NPs or 20 mg/kg/day of PS NPs via oral gavage, using a 1mL syringe and a stainless-steel ball-tipped gavage dosing needle (24 G). The dose was delivered by inserting the ball-tipped needle into the esophagus. Dosing was performed at nearly the same time each day. Each pup was weighed on each dosing day and the appropriate volume of the dosing solution (5 mL/kg) was administered. The vehicle control was deionized water dosed at 5 mL/kg. Since CO_2_ or anesthetic agents can affect neurotransmitters [71,72], pups were euthanized on PND 21 by live decapitation. Dams were euthanized via overexposure to CO_2_. Trunk blood was collected immediately after decapitation and processed into plasma. The liver and brain were collected and weighed. The ratio of liver weight to body weight (liver-to-bw) and the ratio of brain weight to bw (brain-to-bw) were determined. Of note, four brain samples from pups receiving nylon NPs (n = 2 female, n = 2 male) were not included in the weight measurements due to the loss of material during processing. The brain was sectioned longitudinally and placed in separate containers and the right half of the brain was used to evaluate neurotransmitters and metabolites.

#### 2.4.2. Cardiac Assessment

The electrocardiograms (ECGs) were measured non-invasively with ECGenie (Mouse Specific, Inc., Framingham, MA, USA) for nine male and eight or nine female pups randomly selected from each of the three litters for each dosing group at PND 20, using a similar method as previously described [30,31]. The cardiac electrical activity was recorded through the animals’ paws via a shielded acquisition platform and data were acquired with LabChart 8 software (ADInstruments, Dunedin, New Zealand). The awake, unrestrained pups were placed on the platform to acclimate prior to recording the ECGs. Several good-quality ECG sequences were analyzed for each pup and the mean echocardiographic parameters were calculated.

#### 2.4.3. Quantification of Neurotransmitters and Related Metabolites in Brain Tissue

Eight males and eight females from each dosing group were randomly selected for the analysis of neurotransmitters and related metabolites in the brain. As previously described [30,31], ultra-high-pressure liquid chromatography (UPLC) coupled with electrochemical detection (ECD) was used to quantity the neurotransmitter DA and related metabolites DOPAC, HVA, and NE (also called noradrenaline (NA) or noradrenalin), as well as neurotransmitter 5-HT and related metabolite 5-HIAA. Briefly, the right halves of the brains of all pups were prepared in tissue buffer (0.05 M Na_2_HPO_4_, 0.03 M citric acid, and 2 mM ascorbic acid, pH 3). The internal standard solution comprised DHBA dissolved in a tissue buffer at a concentration of 200 ng/mL. To each sample, the solution of internal standard was added at a ratio of 5 mL per g of brain tissue. To extract neurotransmitters/metabolites, ten stainless steel grinding balls (2.8 mm diameter) were added to each brain tissue sample and homogenized with two 30-s cycles on a Geno/Grinder 2010 (SPEX SamplePrep, Metuchen, NJ, USA). After homogenization, samples were centrifuged at 3500× *g* for 10 min at 4 °C, and aliquots of the supernatant were collected and filtered (Ultrafree^®^-MC 0.45 μm Polyvinylidene Fluoride (PVDF); Merck Millipore Ltd., Tullagreen Carrigtwohill, Co., Cork, Ireland). The analysis of processed samples (10 µL aliquot) occurred using a Luna Omega 1.6 μm Polar C18, 2.1 × 150 mm column heated to 32 °C (Phenomenex, Torrance, CA, USA) coupled to a LPG-3400RS pump, WPS-3000TBRS autosampler, and a 5600A CoulArray electrochemical detector (Thermo Scientific, Waltham, MA, USA). The mobile phase comprised 50 mM sodium phosphate, 47 mM citric acid, 0.14 mM EDTA, 0.64 mM octanesulfonic acid, and 5% methanol, at a flow rate of 0.4 mL/min. The detector was set to sequentially deliver potentials of −150 mV, 150 mV, 400 mV, and 600 mV.

#### 2.4.4. Metabolomics Analysis

The AbsoluteIDQ p180 kit (Biocrates Life Sciences AG, Innsbruck, Austria) was used for targeted metabolomics analysis by liquid chromatography–mass spectrometry (LC-MS/MS), as similarly published [73,74]. The kit evaluates the concentration of 40 acylcarnitines, 21 amino acids, 1 monosaccharide, 90 glycerophospholipids, 15 sphingolipids, and 21 biogenic amines, as well as 44 metabolite ratios. From each dosing group, a 10 µL aliquot of plasma samples from 12 males and 12 females was analyzed at PND 21. For each plate, four reference plasma samples were also included, which comprised pooled plasma from dams not exposed to NPs. LC-MS/MS analysis and flow injection were conducted on an API 4000 triple quad mass spectrometer (AB Sciex, Framingham, MA, USA) coupled with an Agilent 1100 high-performance liquid chromatographer (HPLC) (Agilent Technologies, Palo Alto, CA, USA). All data were processed using Analyst 1.6.2 (AB Sciex, Framingham, MA, USA) and MetIDQ Nitrogen 7 software (Biocrates Life Sciences AG, Innsbruck, Austria). Values below the limit of detection (LOD) were not included in the statistical analysis.

#### 2.4.5. Statistical and Multivariate Analysis

To test statistical differences between dosing groups, an unpaired t-test with Welch’s correction was performed for body weight, brain-to-bw and liver-to-bw ratios, and cardiac function. A one-way ANOVA was performed on data for neurotransmitters and related metabolite concentrations (GraphPad Prism 10.1.2, GraphPad Software, San Diego, CA, USA).

Biocrates MetIDQ Nitrogen 7 with the Biocrates MetIDQ StatPack (Biocrates, Life Sciences AG, Innsbruck, Austria) was used for univariate statistical analysis of plasma metabolite data. A Mann–Whitney U-test was used to test the statistical significance of metabolites between the dosing groups and their corresponding vehicle controls. The metabolite fold-change (FC) between individual metabolites was calculated using the mean. Nominal *p*-values were reported for the comparison between animals exposed to NPs and those exposed to vehicle controls.

A multivariate analysis of the metabolomics data was performed via SIMCA 15.0 (Sartorius Stedim Data Analytics, AB, Umeå, Sweden) to reduce the dimensionality and to visualize the differentiation between dosing groups [75,76]. Before the multivariate data analysis, data were mean-centered and unit variance (UV)-scaled. Unsupervised models were created using principal component analysis (PCA) and the scores plots were reviewed to certify the tight clustering of the quality control (QC) pool samples [77]. Supervised analysis, orthogonal partial least squares discriminate analysis (OPLS-DA), was utilized to determine the key metabolites for differentiating study groups based on variable influence on projection (VIP) scores. A VIP score ≥ 1.0 with a jack-knife confidence interval that did not include 0 was deemed important. The VIP statistic summarizes the importance of the metabolites in differentiating the phenotypic groups [75]. All models used a 7-fold cross-validation to assess the predictive ability of the model (Q2).

Metabolites with VIP ≥ 1.0 and/or *p*-value ≤ 0.05 were identified as key metabolites for differentiating the dosing groups against the controls.

#### 2.4.6. Metabolic Pathway Analysis

To evaluate the potential perturbations in metabolic pathways, the free online MetaboAnalyst 5.0 software was used. Metabolites with *p*-value ≤ 0.05 or VIP ≥ 1.0 in the multivariate and univariant statistical analyses were used as inputs for the MetaboAnalyst 5.0 software. For pathway analysis, the Over-Representation Analysis (ORA) method hypergeometric test was utilized, along with the pathway library for rats (KEGG code rno; *Rattus norvegicus*). For pathway topological analysis, the relative betweenness centrality was selected as a node importance measure. The Metabolite Set Enrichment Analysis (MSEA) was utilized to assess consistent changes among lipids, using the ORA method hypergeometric test and the “Sub-class” metabolite set library, which contains 1072 sub-chemical class metabolite sets or lipid sets, based on chemical structures. A false discovery rate (FDR)-adjusted *p*-value ≤ 0.05 indicated significant pathway perturbation.

## 3. Results

### 3.1. Characterization of NPs

**Figure 1:**
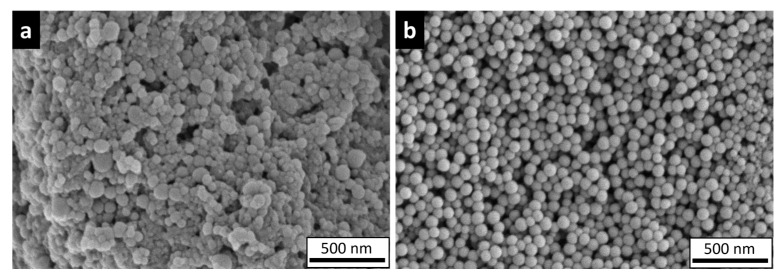
SEM images of (**a**) nylon-11 NPs and (**b**) PS NPs.

Prior to oral administration to rat pups, the physicochemical properties of the nylon-11 NPs and the PS NPs were characterized. Table 1 shows the hydrodynamic diameter (z-average), PDI, and zeta potential for in-house-fabricated nylon-11 NPs and commercially purchased PS NPs. The DLS profiles are shown in Figure S2. Importantly, these properties were analyzed after passing NPs (4mg/mL) through a 24 G needle to ensure the characterization represented the materials tested in vivo. The zeta potential of the NPs suspended in deionized (DI) water at 40 ± 1.5 mV for nylon NPs and −66 ± 2.2 mV for PS NPs supports the observed colloidal stability of the nanoplastics. The endotoxin levels for these NPs (Table 1) are below the limit set by the United States Food and Drug Administration (FDA) for sterile water used for injection [78]. The SEM images reveal a spherical morphology for PS NPs and a slightly more irregular spherical shape for nylon-11 NPs (Figure 1).

### 3.2. Oral Exposure of NPs to Rats

#### 3.2.1. Ratio of Organ-to-BW

**Figure 2:**
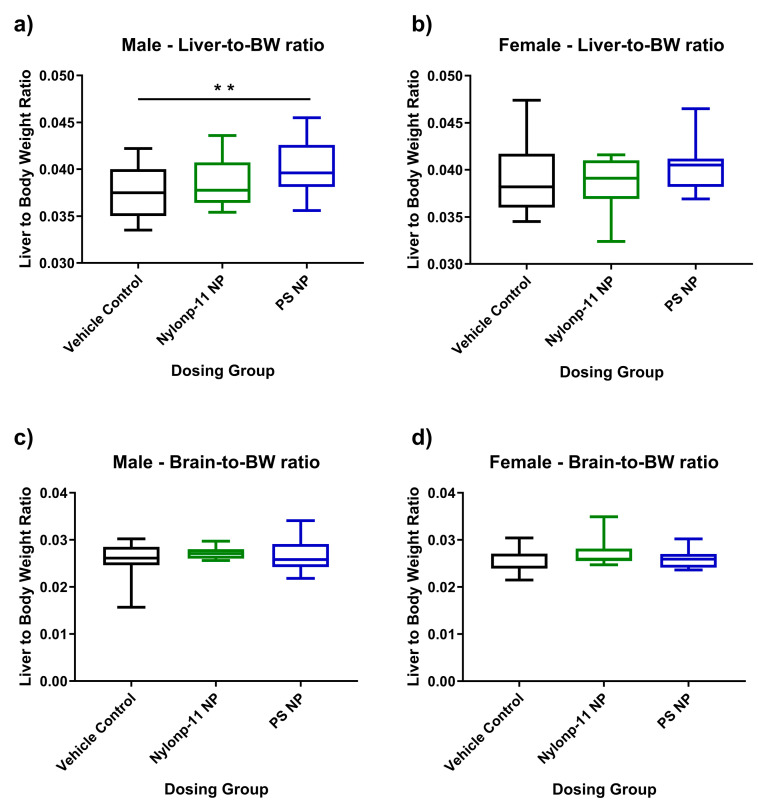
The liver-to-bw ratios for (**a**) males and (**b**) females and the brain-to-bw ratios for (**c**) males and (**d**) females. Statistical analyses were conducted using unpaired t-test with Welch’s correction (** *p* = 0.015).

The body weight was measured for all rat pups between PND 7 and PND 21 and showed no difference between animals that received vehicle controls or NPs (Figure S3). Of note, one male pup that received nylon NPs was euthanized on PND 10 due to low weight gain and was not included in the analysis of body or organ weights. At PND 21, the organ-to-bw ratio was measured for male and female pups dosed between PND 7–10 with nylon-11 NPs, PS NPs, or vehicle controls. The liver-to-bw ratio increased slightly, but significantly (*p* = 0.015), for male pups dosed with PS NPs compared to vehicle controls (Figure 2a). No significant changes in the liver-to-bw ratio occurred in female pups, showing *p* values > 0.05 (Figure 2b). Likewise, no changes in the brain-to-bw ratio were observed in both male and female pups dosed with nylon-NPs or PS NPs (Figure 2c,d).

#### 3.2.2. Cardiac Assessment

The ECGs of pups that were unrestrained and awake were collected at PND 20 and showed no significant differences for male or female animals dosed with the vehicle control, nylon-11 NPs, or PS NPs between PND 7–10 (Figure S4). Despite the lack of differences here, future studies are needed to consider the cardiac effects of NPs with other characteristics, such as compositions and shapes.

#### 3.2.3. Concentrations of Neurotransmitters and Related Metabolites in Brain Tissue

At PND 21, two monoamine neurotransmitters and four metabolites related to memory, emotion, depression, anxiety, and neuroendocrine function were quantified: the neurotransmitter DA and its metabolites DOPAC, HVA, and NE, as well as the neurotransmitter 5-HT and its primary metabolite 5-HIAA. Across all groups, no significant changes occurred in the concentration of these markers after the oral administration of NPs, as compared to the vehicle control (Figures S5 and S6).

#### 3.2.4. Metabolomics Analysis of Plasma

**Figure 3:**
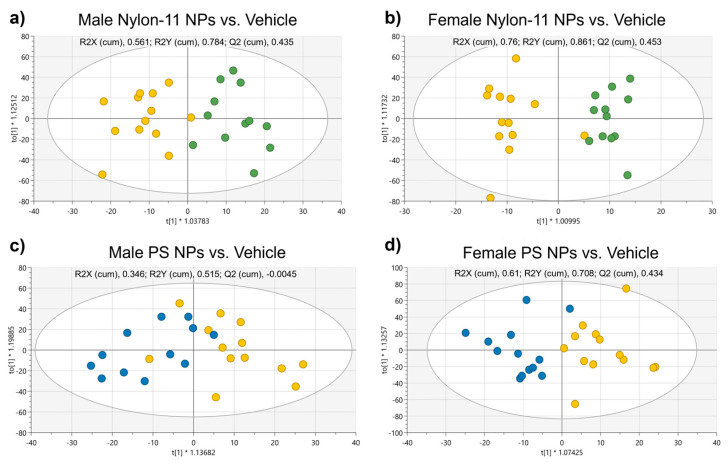
OPLS-DA score plots (R2X, R2Y, and Q2 values of model quality) differentiating male (**a**,**c**) and female (**b**,**d**) rat pups orally administered either nylon-11 NPs (green circles), PS NPs (blue circles) or vehicle control (yellow circles). Rat pups received four daily consecutive doses between PND 7–10 and plasma metabolites were measured on PND 21.

Metabolomics analysis of plasma collected at PND 21 was performed by quantifying a total of 186 metabolites: 40 acylcarnitines, 21 amino acids, 1 monosaccharide, 90 glycerophospholipids, 15 sphingolipids, and 21 biogenic amines. Additionally, 44 metabolism indicators (sums and ratios of selected metabolites) were calculated. A supervised orthogonal partial least squares discriminate analysis (OPLS-DA) was conducted using the metabolites only. For both male and female rat pups, the OPLS-DA score plots showed some differentiation between groups dosed with nylon-11 NPs and vehicle controls (Figure 3a,b). Similarly, a separation of metabolite groupings existed between animals dosed with PS NPs and vehicle controls (Figure 3c,d). Overall, the OPLS-DA plots showed separation between metabolite groupings between rat pups exposed to NPs and vehicle controls, particularly for female animals exposed to nylon-11 NPs (Figure 3b); however, some overlap between the two groups existed. Metabolites with *p* value ≤ 0.05 and/or VIP ≥ 1 in the OPLS-DA for all rat pups are shown in Table S1.

To assess potential perturbations in metabolic pathways after exposure to NPs, analysis was performed separately on small-molecule metabolites and lipid metabolites using the MetaboAnalyst software. Results showed perturbations in the metabolic pathway for aminoacyl-tRNA biosynthesis in male and female pups after exposure to nylon-11 NPs as well as for female pups after exposure to PS NPs (Table 2). Other perturbed metabolic pathways included those associated with amino acid biosynthesis and sphingolipid metabolism. Additionally, enrichment analysis showed perturbations in the pathways for the lipid subclass after exposure to NPs. Lipid subclasses, most notably 1-alkyl,2-acylglycerophosphocholines, diacylglycerophosphocholines, and phosphoshingolipids, were significantly changed for nearly all dosing groups (Table 3).

## 4. Discussion

The existence of MPs and NPs in food and beverages raises fundamental questions about their potential effects on human health. MPs and NPs likely exhibit a wide range of physicochemical properties (e.g., compositions, sizes, shapes), which presents challenges when attempting to unravel how each feature influences biological systems and ultimately human health. To date, the primary focus on using PS nanoparticles for studying effects in biological systems likely stems from the commercial availability of these materials. Currently, nanoparticles comprising many commodity plastics are not readily available for purchase and in certain cases require the development of new fabrication protocols. This study aimed to broaden the understanding of how NPs with polymeric compositions alternative to PS could affect biological systems by using nylon-11 NPs fabricated in-house alongside commercially available PS NPs with similar size distributions. NPs with well-characterized properties are advantageous for exposure studies by narrowing the number of physicochemical variables that can affect biological outcomes.

Both formulations of NPs used in this rat study exhibited an overall spherical morphology and narrow size distribution. The slightly irregular morphology of the nylon-11 NPs may result from the processing method and the material [79]. After fabrication, the nylon-11 NPs did not aggregate when suspended in DI water and showed a PDI of 0.18 ± 0.03. Likewise, the commercial PS NPs also remained in suspension with a PDI of 0.08 ± 0.01. These PDI values are considered low for nanoparticle populations, such as those developed for drug delivery systems [80,81]. The zeta potential of the NPs, as measured in DI water, was 40 ± 1.5 mV and −66 ± 2.2 mV for nylon-11 NPs and PS NPs, respectively. As previously reported, PS nanoparticles can exhibit a net negative charge at a neutral pH resulting from negative groups on the surface from initiators [82,83,84,85]. The positive zeta potential of the nylon-11 NPs likely originates from the amide groups, as the charge density of polyamides results from the ionization of the carboxylic and amine groups in the polymer [86].

With a focus on early-life, this exploratory in vivo study examined the potential effects on rat pups from oral exposure to NPs by measuring biological metrics: body and organ weight, cardiac output, and biochemical profiles. Nylon-11 NPs and PS NPs were administered orally (20 mg/kg/day) to rat pups between PND 7–10 and subsequently monitored through PND 21. Throughout the study, the total body weight of the pups increased but did not show statistical differences between animals that were exposed to NPs and those exposed to the vehicle control (Figure S3). For organ weight, no differences were observed for female rat pups with and without exposure to NPs. Slight differences, however, were observed in the liver-to-bw ratio of males exposed to PS NPs (Figure 2a). Changes in organ weight following exposure to a chemical or material are recognized as an indicator of toxicity [87,88], with numerous studies using this measurement to assess outcomes after exposure to nanomaterials. For example, administration of nickel oxide (NiO) nanoparticles intratracheally to adult rats resulted in an increase in liver wet weight and coefficient of body weight, suggesting potential liver damage [89]. For early-life exposures, orally administered aluminum oxide (AlO_3_) nanoparticles to male rat pups between PND 17–20 resulted in an increased liver-to-body weight ratio [31]. A recent study showed that intraperitoneal administration of PS NPs (20 nm diameter) in adult mice induced necroptosis and promoted acute liver injury [90]. Here, the slight change in the liver-to-bw ratio from exposure to PS NPs requires further investigation to ascertain the potential impacts on hepatic health.

In addition to body weight, the heart rates of the rat pups were measured at PND 20. The ECGs of the pups showed no significant differences for animals dosed with the vehicle control, nylon-11 NPs, or PS NPs (Figure S4). Similar findings exist for other nanoparticles, including a report showing that Al_2_O_3_ NP administered to rat pups at PND 17–20 had no significant impacts on cardiac performance [31]. Interestingly, another study found significant changes in cardiac performance in female rate pups exposed to titanium dioxide (TiO_2_) nanoparticles at specific times (i.e., between PND 7–10 and PND 17–20), suggesting the importance of sensitive timeframes of exposure [30]. Here, the heart rates were measured at PND 20, but future studies might require evaluation at different timepoints to ascertain potential effects. To further evaluate the effects of NPs on brain biochemistry, two monoamine neurotransmitters and four related metabolites were quantified at PND 21: neurotransmitter DA and related metabolites DOPAC, HVA, and NE, as well as neurotransmitter 5-HT and related metabolite 5-HIAA. The selection of these analytes was based on the association of these compounds with memory, emotion, depression, anxiety, and neuroendocrine function. For both sexes, no discernable differences existed in the quantity of neurotransmitters between rat pups exposed to NPs and those exposed to the vehicle control (Figures S5 and S6).

As a selective barrier, the intestinal tract regulates nutrient transport while also hosting the gut microbiome and guiding bidirectional gut–brain interactions [91,92]. Environmental stressors during early-life can disrupt intestinal permeability and microbiota [93], which could lead to effects later in life [94]. In this study, metabolomic analysis of plasma collected at PND 21 showed some changes in amino acids and glycerophospholipids (Table S1), suggesting possible influences on nutrient uptake and metabolism. For instance, amino acid biosynthesis (arginine, valine, leucine, isoleucine) was identified as a perturbed metabolic pathway in female pups exposed to NPs (Table 2). Additionally, aminoacyl-tRNA biosynthesis was identified as another metabolic pathway perturbed after oral administration of nylon-11 NPs (male and female pups) and PS NPs (female pups) (Table 2). As a vital mechanism for normal growth and maintaining the fidelity of protein production, aminoacyl-tRNA synthesis entails the 3’esterification with an amino acid [95]. The noncanonical functions of aminoacyl-tRNA synthetases include gene transcription, angiogenesis, translation, and RNA splicing [96].

Another group of metabolites that was altered by the oral administration of NPs included glycerophospholipids, particularly the subgroup of phosphatidylcholines and lysophosphatidylcholines (Table 3 and Table S1). Similar alterations in glycerophospholipids were observed after the oral administration of TiO_2_ nanoparticles early in life [30]. As a main component of biological membranes and abundantly present in brain tissue, glycerophospholipids participate in various key metabolic, neurological, and intracellular signaling processes [97,98]. Phosphatidylcholine is a key constituent of cellular membranes in mammalian cells and is needed for cell division and growth, particularly in the brain during early-life [99]. Moreover, alterations in the metabolism, signaling, and transport of phospholipids have been associated with certain neurological conditions, such as dementia, schizophrenia, and Parkinson’s disease [100]. Further research is required to understand the short- and long-term consequences of neurological development and metabolism after oral exposure to NPs.

Nylon materials are utilized for biomedical applications including catheter balloons, dentures, tissue engineering, and sutures [58,66]. One example includes a study that used piezoelectric nylon-11 nanoparticles to promote osteogenic differentiation, showing cytocompatibility in dental pulp stem cells [66]. Nylon-11 materials are also produced commercially as Arkema Rilsan^®^ Med for catheters and medical tubing [101]. Such nylon-based medical devices or other clinical form factors, however, are held to strict, controlled standards from regulatory bodies to guide product specifications. Conversely, unintentional exposure to nanoscale nylon particles from the environment will require additional considerations, including exposure to a myriad of physicochemical properties (sizes, shapes, chemical/biological hitchhikers). Similarly, PS is also used for biomedical applications, notably as a material for laboratory consumables (Petri dishes, pipettes). For PS NPs, a variety of studies have evaluated the potential effects of oral exposure in rat models. For instance, daily oral exposure to PS NPs in adult rats (38.92 nm hydrodynamic diameter) for 35 days at doses up to 10 mg/kg/day did not result in any significant behavioral effects but did show subtle and transient neurobehavioral changes [44]. In another study, oral dosing of PS particles of up to 50mg/kd/d in rats (500 nm diameter) showed an upregulation of metabolites with antioxidant and anti-inflammatory effects [102].

Taken together, this study demonstrated some differences in organ weight and biochemical profiles between rat pups exposed to NPs and those exposed to vehicle controls. However, additional studies are needed to fully ascertain the reason for these differences. It is also important to highlight a limitation of this study, namely the narrow number of variables in the NP formulations, which cannot represent the significance of the potential exposure to NPs in the environment. For example, we only evaluated a single oral dose of two different formulations of NPs (spherical, defined size distribution) at 20 mg/kg/day within a defined timeframe (daily between PND 7–10). Although this study is informative, additional work is required to fully ascertain the effects of chronic exposure over expanded timepoints and dose ranges for these formulations of NPs. Moreover, additional studies are needed to address NPs with other physicochemical properties that more fully represent the diversity of potential exposures. For example, many commercial plastic formulations include additives such as plasticizers, colorants, and fillers. Compounding these diverse properties is the potential association of NPs and MPs with the food matrix, which could also introduce variations in biological responses. Given these immense variables, new streamlined approaches are critical for exposure studies, including the use of relevant in vitro toxicology models. The evaluation of NPs with established characteristics, such as the nylon NPs and PS NPs used in this study, can advance the understanding of how the individual features of NPs affect biological systems and ultimately human health.

## 5. Conclusions

The possible health risks of oral ingestion of NPs and MPs are largely unknown, which has spurred efforts to evaluate biological responses after exposure to these materials. In this study, a key goal was to evaluate how early-life exposure to thoroughly characterized NPs affects key biological metrics in male and female rat pups. To the best of our knowledge, this is the first study to explore the biological impacts of oral exposure to nylon NPs early in life. Animals receiving an oral dose (20 mg/kg/day) of NPs between PND 7-10 showed differences in organ weight and biochemical profiles, as compared to animals receiving vehicle controls. In particular, alterations in metabolites such as amino acids and glycerophospholipids were observed in the rat pups orally exposed to NPs. Moreover, the metabolite plasma profile was altered after oral administration of NPs, showing the perturbation of pathways involved in amino acid and lipid metabolism. No differences in the cardiac function or quantity of brain neurotransmitters and related metabolites were observed between pups exposed to NPs and vehicle controls. A critical need remains for the continued exploration of how oral exposure to small-scale plastics affects human health.

## 6. Patents

The authors (L.M.J., A.K., N.P.M.) are named as inventors on pending patent applications filed by RTI International. The institution has a policy that grants a portion of any royalties received to named inventors.

## Figures and Tables

**Table 1:** Properties of NPs used for the in vivo study.

N	Hydrodynamic Diameter (nm)	PDI	Zeta Potential (mV)	Endotoxin (EU/mL)
Nylon-11	114 ± 2	0.18 ± 0.03	40 ± 1.5	0.054
PS	85 ± 1	0.08 ± 0.01	−66 ± 2.2	0.048

**Table 2:** Pathway analysis results (FDR correct *p* values) using MetaboAnalyst showing enriched metabolic pathways perturbed upon exposure to NPs. FDR correct *p* values ≤ 0.05 indicates a significant perturbation in a pathway.

Metabolic Pathway	Nylon-11 NPs		PS NPs	
	Male	Female	Male	Female
Aminoacyl-tRNA biosynthesis	2.63 × 10^−6^	1.16 × 10^−13^	-	5.14 × 10^−7^
Arginine biosynthesis	-	-	-	3.34 × 10^−4^
Sphingolipid metabolism	-	-	4.61 × 10^−2^	-
Valine, leucine, and isoleucine biosynthesis	-	2.7 × 10^−3^	-	-

FDR corrected *p* values > 0.05 indicated by ‘-’.

**Table 3:** Results (FDR correct *p* values) of Metabolite Set Enrichment Analysis using MetaboAnalyst showing consistent changes among lipids. FDR correct *p*-values ≤ 0.05 indicates a significant perturbation in a pathway.

Lipid Subclass	Nylon-11 NPs		PS NPs	
	Male	Female	Male	Female
1-alkyl,2-acylglycerophosphocholines	2.95 × 10^−8^	-	6.37 × 10^−3^	3.74 × 10^−10^
Diacylglycerophosphocholines	3.62 × 10^−49^	2.36 × 10^−2^	1.23 × 10^−5^	3.85 × 10^−2^
Fatty acyl carnitines	-	-	-	7.73 × 10^−3^
Lysophosphatidylcholines	-	-	-	7.73 × 10^−3^
Phosphosphingolipids	2.95 × 10^−3^	-	3.94 × 10^−7^	-
Sphingomyelins	-	-	5.56 × 10^−3^	-

FDR corrected *p* values > 0.05 indicated by ‘-’.

## Data Availability

The original contributions presented in this study are included in the article and supplementary materials. Further inquiries can be directed at the corresponding author.

## Supplementary Materials

The following supporting information can be downloaded at: https://www.mdpi.com/article/10.3390/nano15060465/s1, Figure S1. Design for the in vivo study of nylon-11 NPs and PS NPs in Sprague Dawley rats; Figure S2. DLS profiles for Nylon-NPs and PS-NPs; Figure S3. Body weight of male and female rats between PND 7 and PND 21 that received orally administered nylon-11 NPs, PS NPs, or vehicle control daily between PND 7–10. No differences existed between vehicle controls and NPs (unpaired t-test with Welch’s correction *p* > 0.05); Figure S4. Heart rate of male and female rats measured on PND 20; Figure S5. Quantity of neurotransmitter (a) DA and related metabolites (b) DOPAC, (c) HVA, and (d) NE, as well as neurotransmitter (e) 5-HT and related metabolite (f) 5-HIAA in the brain of male rat pups (n = 8 for nylon-11 dose group, n = 8 for PS dose group, n = 7 for vehicle dose group) at PND 21; Figure S6. Quantity of neurotransmitter (a) DA and related metabolites (b) DOPAC, (c) HVA, and (d) NE, as well as neurotransmitter (e) 5-HT and related metabolite (f) 5-HIAA in the brain of female rat pups (n = 8 per dose group) at PND 21; Table S1. Metabolites in plasma collected from rat pups with significant *p* value ≤ 0.05 and/or VIP ≥ 1.0 with an S.E. less than mean. Metabolites with a *p* value ≤ 0.1 are also shown.
