# Supplementary material for: Oral Exposure to Nylon-11 and Polystyrene Nanoplastics During Early-Life in Rats
Source: Nanomaterials (Basel). 2025 Mar 19;15(6):465. doi: 10.3390/nano15060465 (PMC11944792; doi:10.3390/nano15060465)
Supplement: Supplementary file 1 [file nanomaterials-15-00465-s001.zip › nanomaterials-3494082- supplementary-AE provided.pdf]

nanomaterials

Supplementary Materials

Oral Exposure to Nylon-11 and Polystyrene Nanoplastics During Early Life in Rats

Ninell P. Mortensen, Maria Moreno Caffaro, Archana Krovi, Jean Kim, Scott L. Watson, Rodney W. Snyder, Purvi R. Patel, Timothy R. Fennell and Leah M. Johnson*

RTI International, 3040 E. Cornwallis Road, Research Triangle Park, Durham, NC 27709, USA
* Correspondence: leahjohnson@rti.org

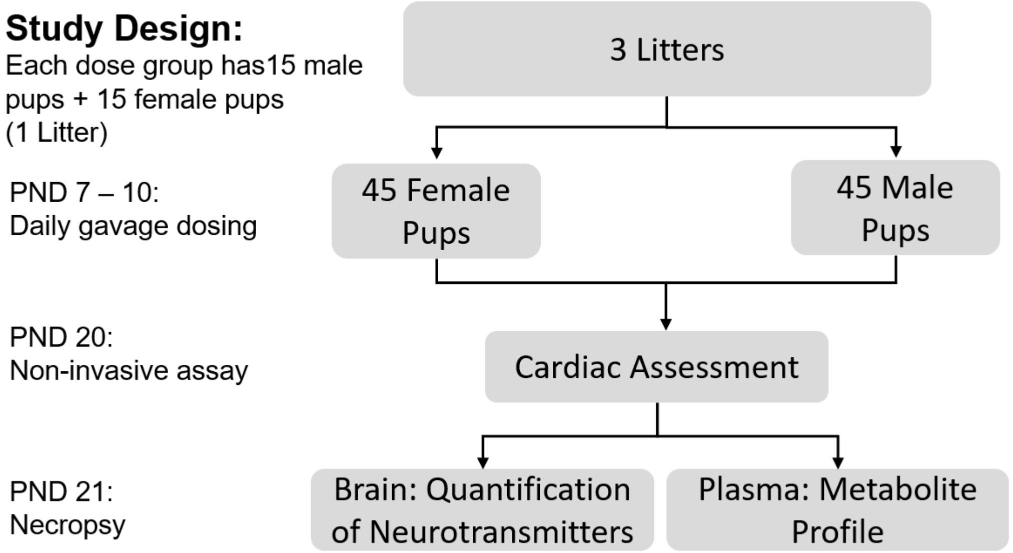

Figure S1. Design for the in vivo study of nylon-11 NPs and PS NPs in Sprague Dawley rats.

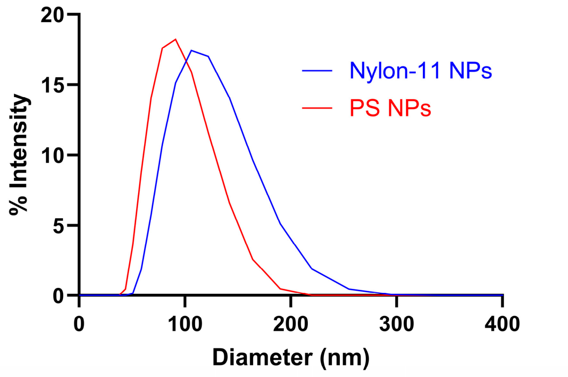

Figure S2. DLS profiles for Nylon-NPs and PS-NPs.

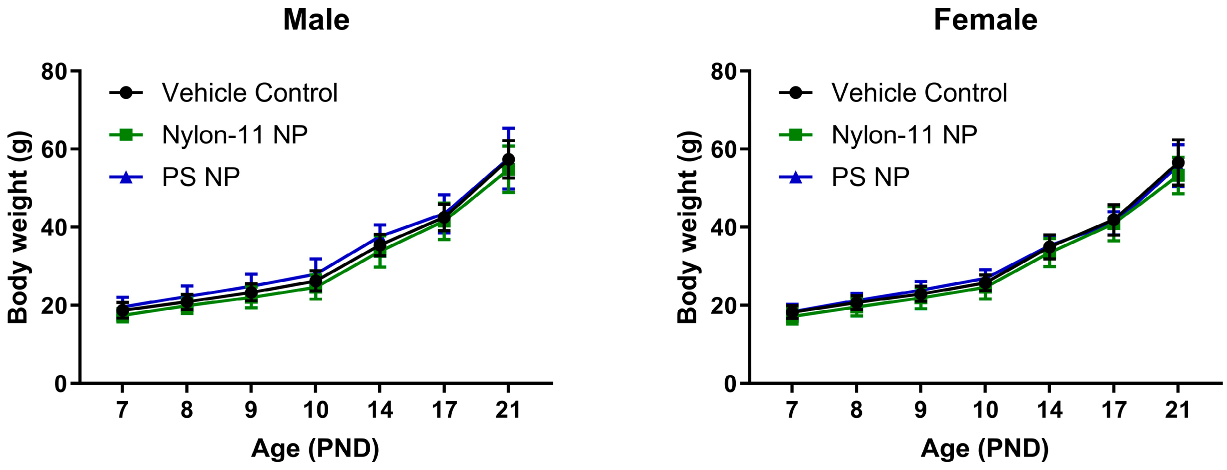

Figure S3. Body weight of male and female rats between PND 7 and PND 21 that received orally administered nylon-11 NPs, PS NPs, or vehicle control daily between PND 7-10. No differences existed between vehicle controls and NPs (unpaired t test with Welch's correction p>0.05).

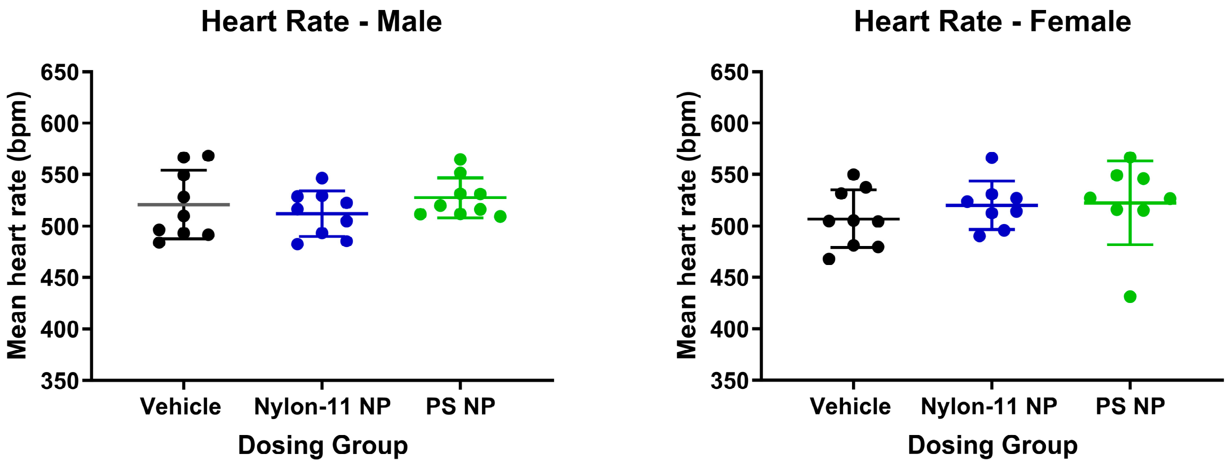

Figure S4. Heart rate of male and female rats measured on PND 20.

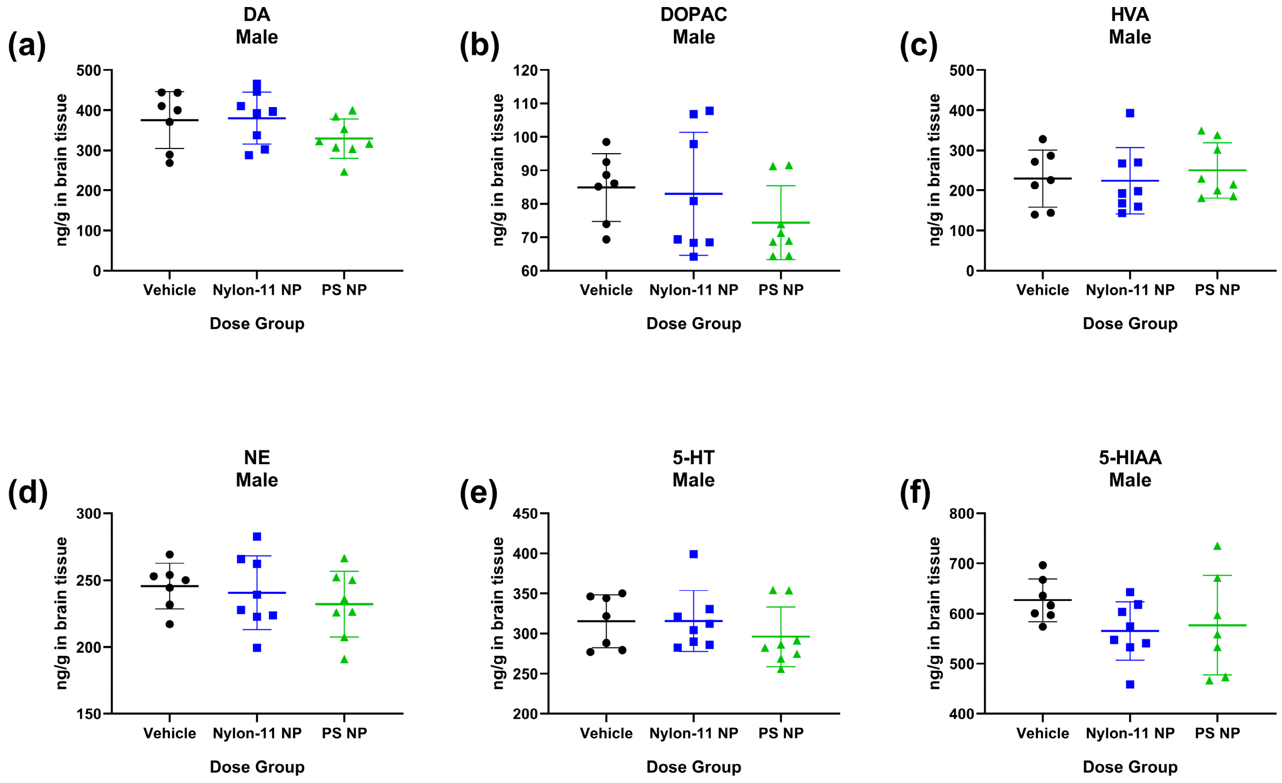

Figure S5. Quantity of neurotransmitter (**a**) DA and related metabolites (**b**) DOPAC, (**c**) HVA, and (**d**) NE, as well as neurotransmitter (**e**) 5-HT and related metabolite (**f**) 5-HIAA in the brain of male rat pups (n=8 for nylon-11 dose group, n=8 for PS dose group, n=7 for vehicle dose group) at PND 21.

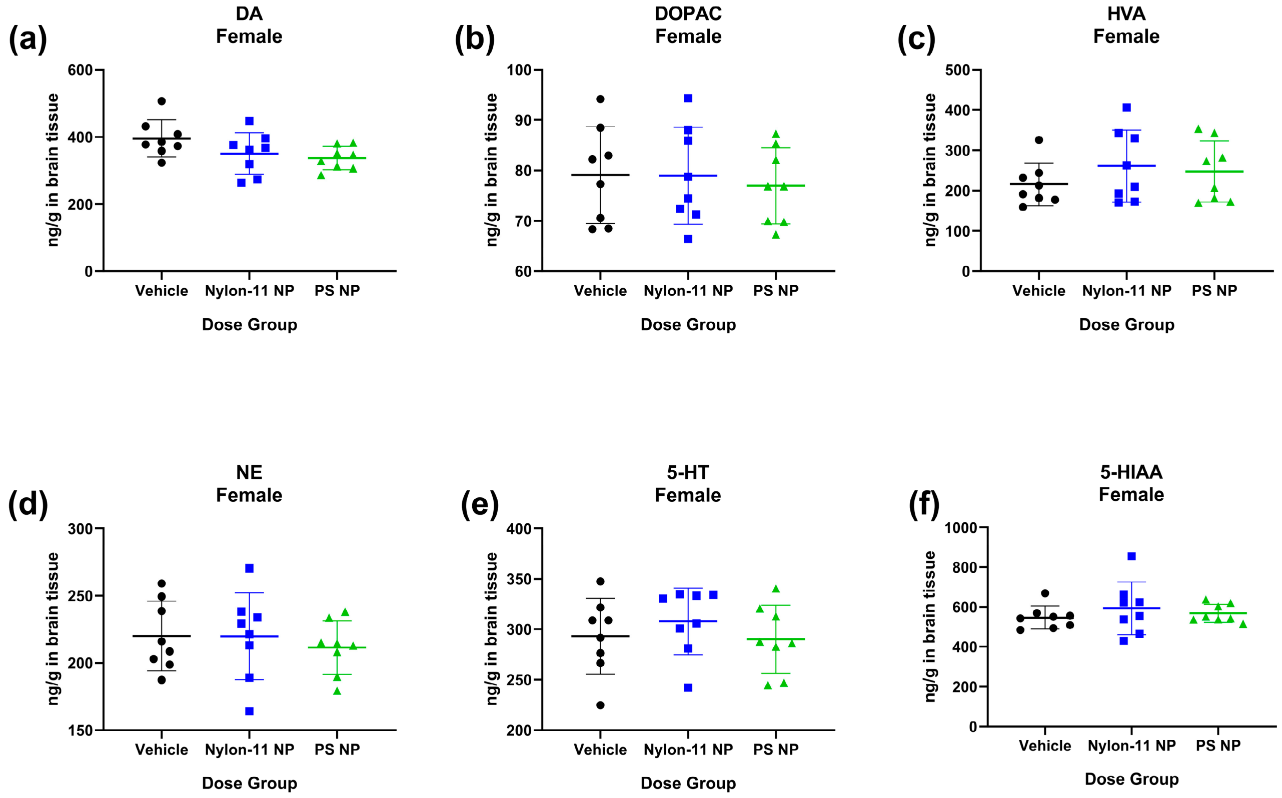

Figure S6. Quantity of neurotransmitter (**a**) DA and related metabolites (**b**) DOPAC, (**c**) HVA, and (**d**) NE, as well as neurotransmitter (**e**) 5-HT and related metabolite (**f**) 5-HIAA in the brain of female rat pups (n=8 per dose group) at PND 21.

Table S1. Metabolites in plasma collected from rat pups with significant p-value ≤ 0.05 and/or VIP ≥ 1.0 with an S.E. less than mean. Metabolites with a p-value ≤ 0.1 are also shown.

	Male Nylon-11 NPs			Female Nylon-11 NPs			Male PS NPs			Female PS NPs		
**Metabolite**	**p-value**	**VIP**	**fold change**	**p-value**	**VIP**	**fold change**	**p-value**	**VIP**	**fold change**	**p-value**	**VIP**	**fold change**
**Acylcarnitines**												
Carnitine	-	-	-	0.05	1.00	0.97	-	-	-	-	-	-
Tetradecenoyl-carnitine	-	-	-	0.04	0.02	0.95	-	-	-	-	-	-
Hexadecadienyl-carnitine	-	-	-	-	-	-	-	-	-	0.04	0.03	1.19
Hexanoylcarnitine	-	-	-	-	-	-	-	-	-	0.04	0.05	1.04
**Amino Acids**												
Alanine	0.01	5.19	0.81	0.79	2.24	0.91	-	-	-	-	-	-
Arginine	-	-	-	0.96	1.19	1.14	-	-	-	-	-	-
Asparagine	0.13	1.68	0.84	-	-	-	-	-	-	-	-	-
Aspartate	-	-	-	-	-	-	-	-	-	0.04	1.93	1.36
Citrulline	0.74	1.16	0.89	-	-	-	-	-	-	0.14	1.71	0.97
Glutamine	0.46	3.69	1.01	0.20	4.69	1.06	-	-	-	-	-	-
Glutamate	-	-	-	-	-	-	-	-	-	0.04	3.16	1.25
Glycine	0.04	5.55	0.87	0.91	1.94	1.06	-	-	-	0.68	2.32	1.02
Histidine	0.51	1.04	0.88	0.70	1.10	0.94	-	-	-	-	-	-
Isoleucine	_	_	_	0.14	1.62	1.18	-	-	-	-	-	-
Leucine	-	-	-	0.16	2.89	1.18	-	-	-	0.82	1.45	1.15

Lysine	-	-	-	0.05	6.18	1.01	-	-	-	0.06	5.42	1.33
Methionine	-	-	-	0.02	1.60	0.99	-	-	-	0.06	1.82	0.88
Ornithine	-	-	-	-	-	-	-	-	-	0.16	1.71	0.95
Proline	0.09	2.31	0.83	0.83	1.72	0.96	-	-	-	-	-	-
Serine	0.85	2.95	0.80	-	-	-	0.08	4.78	1.02	0.18	2.98	1.13
Tryptophan	-	-	-	-	-	-	-	-	-	0.09	1.66	0.95
Tyrosine	-	-	-	0.76	1.34	0.91	-	-	-	-	-	-
Valine	-	-	-	0.19	1.70	1.16	-	-	-	-	-	-
**Biogenic Amines**												
Spermine	0.05	0.34	0.79	-	-	-	-	-	-	-	-	-
Taurine	0.04		0.76	-	-	-	-	-	-	0.02	NA	0.83
Methionine Sulfoxide	-	-	-	-	-	-	-	-	-	0.05	0.55	0.94
cis-4-Hydroxy-proline	-	-	-	-	-	-	0.03	0.04	0.77	-	-	-
trans-4-Hydroxy-proline	-	-	-	-	-	-	-	-	-	0.59	1.02	1.05
**Glycerophospholipids (Lysophosphatidylcholines [lysoPC]; Phosphatidylcholines [PC]***												
lysoPC a C14:0	-	-	-	-	-	-	-	-	-	0.04	0.38	0.96
lysoPC a C17:0	-	-	-	-	-	-	-	-	-	0.03	0.23	1.09
lysoPC a C18:0	0.04	1.28	1.10	0.13	1.04	1.15	-	-	-	-	-	-
PC aa C28:1	-	-	-	-	-	-	0.03	0.10	1.06	-	-	-
PC aa C36:5	-	-	-	0.04	0.40	1.03	-	-	-	0.01	0.50	0.89
PC aa C38:0	0.01	0.26	1.12	-	-	-	-	-	-	-	-	-
PC aa C38:1	0.03	0.14	1.33	-	-	-	-	-	-	-	-	-
PC aa C38:4	0.02	2.46	1.12	-	-	-	-	-	-	-	-	-
PC aa C38:6	0.03	1.48	1.09	-	-	-	-	-	-	-	-	-

PC aa C40:4	0.04	0.22	1.21	-	-	-	-	-	-	-	-	-
PC aa C40:5	-	-	-	-	-	-	0.01	0.68	0.97	-	-	-
PC aa C40:6	0.01	1.32	1.18	-	-	-	-	-	-	-	-	-
PC aa C42:6	-	-	-	-	-	-	0.00	0.16	1.01	-	-	-
PC ae C30:0	-	-	-	0.03	0.09	0.88	-	-	-	-	-	-
PC ae C36:1	-	-	-	-	-	-	-	-	-	0.04	0.21	0.99
PC ae C36:3	-	-	-	-	-	-	-	-	-	0.05	0.23	1.05
PC ae C38:2	0.03	0.26	1.22	-	-	-	-	-	-	-	-	-
PC ae C38:4	0.00	0.42	1.22	-	-	-	-	-	-	0.04	0.37	1.04
PC ae C38:5	0.01	0.52	1.10	-	-	-	-	-	-	-	-	-
PC ae C38:6	0.03	0.32	1.09	-	-	-	-	-	-	-	-	-
PC ae C40:3	-	-	-	-	-	-	-	-	-	0.03	0.11	0.91
PC ae C40:5	0.01	0.23	1.18	-	-	-	-	-	-	-	-	-
PC ae C40:6	0.00	0.25	1.22	-	-	-	-	-	-	-	-	-
PC ae C42:1	-	-	-	-	-	-	0.03	0.11	0.98	-	-	-
PC ae C44:4	-	-	-	-	-	-	-	-	-	0.05	0.04	1.14
PC ae C44:5	-	-	-	-	-	-	-	-	-	0.03	0.02	1.02
**Sphingolipids (Sphingomyelins [SM]; Hydroxylated Sphingomyelins [SM (OH)])***												
SM (OH) C14:1	0.00	0.15	1.34	-	-	-	-	-	-	-	-	-
SM (OH) C16:1	-	-	-	0.01	0.19	1.12	-	-	-	-	-	-
SM (OH) C22:1	-	-	-	-	-	-	0.03	0.33	0.98	-	-	-
SM (OH) C22:2	-	-	-	-	-	-	0.00	0.21	0.97	-	-	-
SM (OH) C24:1	0.04	0.08	1.20	-	-	-	-	-	-	-	-	-
SM C20:2	-	-	-	0.03	0.12	1.29	-	-	-	-	-	-
SM C22:3	0.02	0.13	2.84	-	-	-	-	-	-	-	-	-
SM C24:0	-	-	-	-	-	-	0.02	0.54	1.06	-	-	-

SM C24:1	-	-	-	-	-	-	0.04	0.53	1.06	-	-	-
**Sugars**												
Hexoses (including glucose)	-	-	-	0.87	6.43	1.03	-	-	-	0.74	6.88	1.01

*Biocrates nomenclature: 'CX:Y', with X=number of carbon atoms in side chain and Y=number of double bonds in side chain.
